# Correction: Chavda et al. Peptide-Drug Conjugates: A New Hope for Cancer Management. *Molecules* 2022, *27*, 7232

**DOI:** 10.3390/molecules30122579

**Published:** 2025-06-13

**Authors:** Vivek P. Chavda, Hetvi K. Solanki, Majid Davidson, Vasso Apostolopoulos, Joanna Bojarska

**Affiliations:** 1 Department of Pharmaceutics and Pharmaceutical Technology, L M College of Pharmacy, Ahmedabad 380008, Gujarat, India; 2 Institute for Health and Sport, Victoria University, Melbourne, VIC 3030, Australia; majid.hassanzadeganroudsari@vu.edu.au; 3 Immunology Program, Australian Institute for Musculoskeletal Science, Melbourne, VIC 3021, Australia; 4 Institute of General and Ecological Chemistry, Faculty of Chemistry, Lodz University of Technology, 116 Zeromskiego Street, 90-924 Lodz, Poland

## Citation Correction

In the original publication cited above [1], reference 5 (“Chavda, V.P.; Chen, R.; Patel, A.B.; Chen, Z. Phytochemical delivery through Transferosome (Phytosome): An advanced transdermal drug delivery for complementary medicine. *Front. Pharmacol.* **2022**, *13*, 850862”) was incorrectly cited. The citation has now been replaced with the following:

Liu, B.; Zhou, H.; Tan, L.; Siu, K.T.H.; Guan, X.-Y. Exploring treatment options in cancer: Tumor treatment strategies. *Signal Transduct. Target. Ther.* **2024**, *9*, 175. https://doi.org/10.1038/s41392-024-01856-7.

The authors state that the scientific conclusions are unaffected. This correction was approved by the Academic Editor. The original publication has also been updated.
